# The Immunocytochemistry Is a Valuable Tool in the Diagnosis of Papillary Thyroid Cancer in FNA's Using Liquid-Based Cytology

**DOI:** 10.1155/2010/963926

**Published:** 2010-10-27

**Authors:** Kalliopi Pazaitou-Panayiotou, Nikolas Mygdakos, Kyriaki Boglou, Anastasia kiziridou, Alexandra Chrisoulidou, Chariklia Destouni

**Affiliations:** ^1^Department of Endocrinology-Endocrine Oncology, Theagenio Cancer Hospital, 2 Al Simeonidi Street, 54007 Thessaloniki, Greece; ^2^Department of Cytopathology, Theagenio Cancer Hospital, 2 Al Simeonidi Street, 54007 Thessaloniki, Greece; ^3^Department of Histopathology, Theagenio Cancer Hospital, 2 Al Simeonidi Street 54007 Thessaloniki, Greece

## Abstract

*Introduction*. Papillary thyroid carcinoma (PTC) is the most common malignancy of the thyroid. An accurate cytological diagnosis is based on distinctive cytological features in combination with immunocytochemistry. *Methods*. A number of 83 fine needle aspirations, positive for papillary thyroid cancer (44 from thyroid nodules and 39 from cervical lymph nodes), were studied using Thin Layer Cytology. A panel of the immunomarkers Cytokeratin-19, Galectin-3, HBME1, CD-44, CD-56, and E-Cadherin was performed. *Results*. Positive expression of CK-19 was observed in 77 cases (92.7%), of Galectin-3 in 74 cases (89.1%), of HBME1 in 65 (78.3%), and of CD-44 in 72 cases (86.7%). Loss of expression of CD-56 was observed in 80 cases (96.4%) and of E-cadherin in 78 (93.9%). *Conclusions*. Our data suggest that Thin Layer Cytology increases the diagnostic accuracy in papillary carcinoma and seems to be a promising technique for further investigation of thyroid lesions permitting the possibility to use archive material. Positive immunoexpression of CK-19, Galectin-3, HBME-1, and CD-44 improves the diagnostic accuracy of papillary thyroid cancer. Furthermore, loss of E-cadherin and of CD-56 expression is a feature of malignancy.

## 1. Introduction

Papillary thyroid carcinoma (PTC) is the most common malignancy of the thyroid gland, and fine needle aspiration cytology (FNAC) is the only preoperative diagnostic method used in its detection. The cytological diagnosis of PTC is considered particularly reliable and allows for a definitive diagnosis and treatment planning at the time of FNA [1, 2] as it is based on specific cytomorphological characteristics. However, these characteristics can occur in other thyroid lesions, both benign and malignant. In such cases, immunocytochemistry seems to play an important role in order to facilitate the uncertain results and helps to avoid diagnostic pitfalls. 

Although FNAC is widely accepted as the primary diagnostic procedure for thyroid nodules [1], few reports have been published regarding immunocytochemistry mainly on liquid-based Thin Layer technique [3]. According to recent research, several diagnostic and prognostic markers have been proposed in improving the diagnostic accuracy of papillary thyroid cancer such as Cytokeratin-19 (CK-19), HBME-1, Galectin-3, CD44, CD56, E-cadherin, c-erbB-2, p21^cip1^, p27^kip1^, and p16^ink4a^ [4–13].

The purpose of our study was the evaluation of immunochemistry in combination with cytomorphology in the diagnosis of papillary cancer in FNA smears using liquid-based cytology.

## 2. Materials and Methods

Cytopathological files of primary papillary thyroid cancer as well as metastatic ones were reviewed from our cases during the last five years. A total number of 83 FNA cases were included; 44 were from thyroid nodules and 39 from cervical lymph nodes. In addition, 30 benign cases that belonged mainly in thyroid adenomatous nodules were used as control. The majority of the cases concerned ultrasound-guided aspirations of thyroid nodules or lymph nodes performed in collaboration with an experienced endocrinologist and cytopathologist on site. All malignant cases and controls were histologically confirmed. 

The cytological material was prepared using, mainly, liquid-based cytology. The conventional method was used in 13 cases. Immediately after the aspiration, one or two conventional smears were prepared, and the remaining sample was rinsed immediately in a Preservcyt (Cytyc Corporation, Boxborough, MA). A thin evenly dispersed monolayer of cells was dispersed from the filter onto the slide in a cycle of 20 mm in diameter. ThinPrep slides were prepared in the laboratory using the TP2000 instrument, and both conventional and ThinPrep slides were stained with a modified Papanicolaou method. Immunocytochemistry was performed only in slides prepared by thin layer method. All samples were stained in an automated immunostainer (Ventana ES, Ventana medical system, Inc., Tucson, AZ, USA) and the related Ventana reagents were used, using standard manufacturer's instructions. Our panel included Cytokeratin 19 (Dilution 1 : 50, Clone b170, IgG-1, Ylem), Galectin-3 (Dilution 1:50, Clone 9C4, Novocastra), HBME-1 (Dilution 1 : 50, monoclonal HBME, Ylem), CD 44 (Dilution 1:50, Clone DF1485, IgG1, Dako), CD-56 (Dilution 1:50, Clone Moc-1, Dako), and E-cadherin (Dilution 1:25, Clone 36B5, Novocastra). 

According to manufacturer's suggestions, the samples were immersed in a citrate buffer solution and heated for 20 minutes at 350 W. The slides were rinsed in tap water for 5 minutes, and then they were incubated with 3% H_2_O_2_ for 4 minutes to quench the endogenous peroxidase activity. We used the primary antibodies diluted in an appropriate dilution with Ventana antibody diluent in a Venatana user fillable dispenser. A standard avidin-biotin method was applied. The primary antibody was bound by a biotin-conjugated mouse secretory antibody formulation and next an avidin enzyme conjugate bound to the biotin present on the secondary antibody followed. The primary antibody-secondary antibody-avidin enzyme complex was then visualized utilizing a precipitating enzyme product. The staining intensity was assessed in a semiquantitative way, independently by two experienced cytopathologists. A scale from negative (0) to weak (1), moderate (2), and strong (3) was applied to each slide while the pattern of stain was classified as membranous or cytoplasmic, diffuse or nuclear. A positive stain was defined by the presence of either a moderate (2) or a strong stain (3) in at least 10% of tumor cells, and the percentage of the remaining positive cells, was evaluated.

## 3. Statistical Analysis

A standard independent-samples *t*-test was used for the evaluation of differences in the expression between each immunomarker in cancer material and controls, respectively. Calculations were carried out using SPSS version 14.0. Results were considered statistically significant when *P*-value was less than .05.

## 4. Results

**Figure 1:**
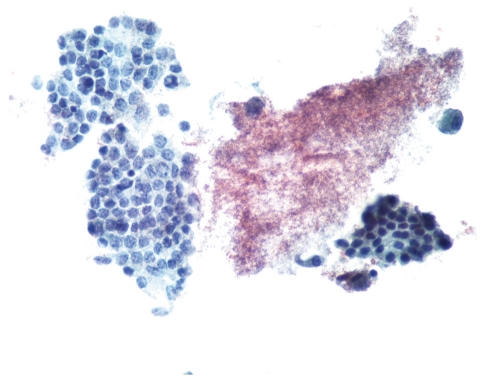
Papillary thyroid carcinoma (left) and normal thyroid tissue (right) (Pap stain ×400).

**Figure 2:**
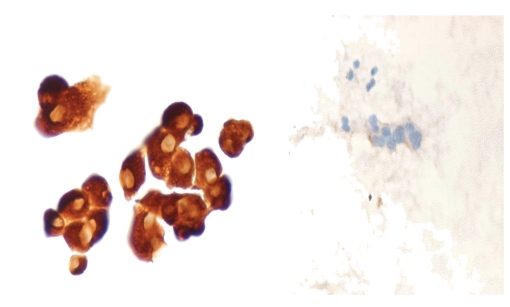
CK-19 expression in a PTC case (left) and negative normal control (right) (ThinPrep ×400).

**Figure 3:**
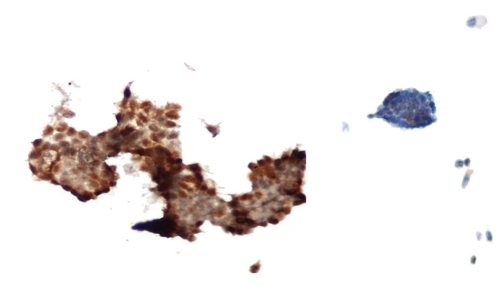
Galectin-3 expression in a PTC case (left) (×400) and negative normal control (right).

**Figure 4:**
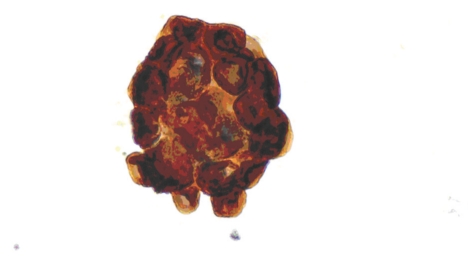
HBME-1 expression in a PTC case (×400).

**Figure 5:**
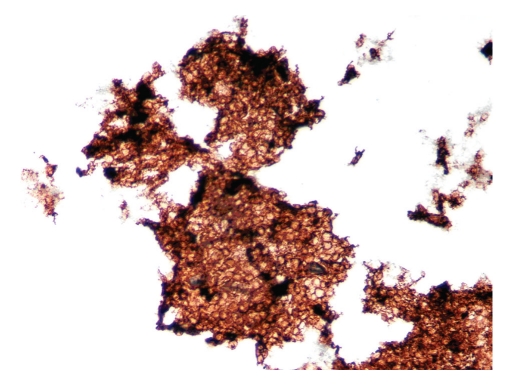
CD-44 expression in a PTC case (×100).

Our cases included 44 FNAs of thyroid nodules, diagnosed as papillary thyroid cancers, and 39 FNAs from cervical lymph nodes with metastases from papillary thyroid cancer too (Figure 1). CK-19 was positive in 77 out of 83 cases (92.7%), with strong diffuse membranous and cytoplasmic staining (Figure 2). Galectin-3 was positive in 74 out of 83 cases (89.1%) with a strong cytoplasmic immune expression (Figure 3). HBME1 showed a predominantly strong membranous pattern and was positive in 65 out of 83 cases (78.3%) (Figure 4). CD-44 was positive in 72 out of 83 cases (86.7%), and the staining was intense membranous and diffuse cytoplasmic (Figure 5). No sample of the control group showed positivity in the above four controlled immunomarkers (Table 1(a)).

A week positive stain to CD-56 was observed in 3 (3.6%) cases, while 80 (96.4%) cases showed loss of expression. E-cadherin was positive in 5 out of 83 (6%) cases, and 78 (93.97%) cases showed loss of expression. All controls retained their normal expression in the above two immunomarkers (Table 1(b)).

**Figure 6:**
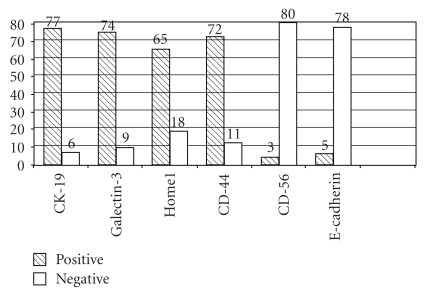
Schematic presentation of the expression of each of the six immunomarkers in our 83 cases. Positive means cases with expression of the immunomarker. Negative means cases with no expression or loss of expression, (in the case of CD-56 and E-Cadherin).

The immunoexpression of all markers was similar in both thyroid and lymph node FNAs. The microscopic evaluation of each immunomarker expression is summarized in Tables 1(a) and 1(b) and Figure 6.

## 5. Discussion

It is well known that FNA is usually the first choice for the preoperative diagnostic evaluation of thyroid nodules in everyday clinical practice [1, 2]. A preoperative accurate diagnosis of papillary thyroid cancer is important in determining the clinical management of these patients. Acquiring a good aspirate is the first step toward a correct diagnosis. The preoperative diagnosis of PTC with FNAc does not present difficulties, as PTC presents distinctive features including nuclear grooves, papillary fronds, monolayered sheets of cells, psammoma bodies, multinucleate giant cells, and intranuclear cytoplasmic inclusions. However, these cytomorphologic criteria are not always possible to observe. Furthermore, follicular and papillary patterns are often overlapping between benign and malignant lesions. For these reasons, diagnostic pitfalls may be noted [14]. Based on our experience and according to the literature data, the follicular variant of papillary carcinoma is thought to be one of the most common causes of false negative cytologic diagnosis of PTC [15]. On the other hand, occult and cystic papillary carcinoma may be a source of error. Therefore, the distinction of true papillary thyroid carcinoma from lesions that share some cytologic features with PTC is of clinical importance. We suggest that immunocytochemistry can play an important role in the differential diagnosis of these uncertain or borderline cases [4, 9, 15].

In this study, we found that CK-19, Galectin-3, CD-44, and HBME1 were highly expressed in papillary carcinomas, a finding that is in agreement with other data reported in the literature [5, 8, 10, 16–19]. Moreover, we demonstrated that the immunoexpression of CD-56 and E-cadherin was absent in almost all cases of this study and this coincides with literature data.

It is reported that CK-19, a cytoskeletal protein, is significantly increased in papillary thyroid carcinoma and is helpful in distinguishing papillary thyroid cancer from benign or other malignant thyroid carcinomas [4]. A strong diffuse or membranous immunoexpression stain, as we have found in our cases, is considered to be in favor of PTC, but focal CK19 staining may be found in benign lesions as well. Galectin-3 is a glucoprotein that plays an important role in organogenesis. It belongs to the family of lectins, is localized mainly in the cytoplasm, and is involved in regulating cell-cell and cell-matrix interactions. Galectin-3 staining is considered positive when cytoplasmic membranous or nuclear staining is present. Many series, as well as the present study, have showed that Galectin-3 is useful as a marker of malignancy in thyroid nodules although some studies have produced conflictive results [20]. The standard form of CD-44, an adhesion molecule, has been associated with extracellular matrix adhesion and lymphocyte homing. Variable expression of CD-44in PTCs has been demonstrated, and these carcinomas were found to express intense cell membrane or diffuse cytoplasmic staining. CD-44 was expressed in 86.7% of our cases, and the staining was intense membranous and diffuse cytoplasmic. HBME1 is a marker of mesothelial cells and is expressed in malignant thyroid follicular tumors. It is recently applied as an immunomarker in PTCs with high expression. However, this positive immunoexpression does not exclusively indicate papillary differentiation [21]. Our results showed that HBME1 is expressed in a high percentage of PTCs cases. 

CD-56, a neural cell adhesion molecule, is present in follicular epithelial cells of normal thyroid. The expression of CD-56 protein was found to be strong within all nonmalignant thyroid cells, but not in cases of PTCs [5]. 96.4% of our cases with PTC showed loss of its expression. On the contrary, all our controls expressed CD-56. E-cadherin, a 120-Kda glucoprotein with a transmembrane domain, is a calcium-dependent homophilic cell adhesion molecule which plays a central role in epithelial integrity, in cell adhesion and differentiation, as well as in the maintenance of cell polarity and tissue architecture. Its impairment correlates with tumor invasion and metastasis [22]. 94% of the cases in this study showed loss of the expression of E-cadherin and all control aspirates expressed it. We suggest that loss of expression of CD-56 and E-cadherin may provide an objective diagnostic tool, and they may be extremely useful in the diagnosis of PTCs, especially in equivocal cases.

Cytologic material used by the conventional method is in most cases unavailable for additional investigation either due to the inadequacy of cellularity or the presence of excess blood mucus or inflammation. This gap is filled by liquid-based cytology and thin layer techniques which may be effective innovations [23]. The quality of the immunocytochemical reaction on the thin layer prepared slides as far as the morphologic details, and the purity of background is considered to be better than conventional smears. Liquid-based cytology offers the possibility of creating archival material and applying new techniques, such as immunocytochemistry in the same sample, and this is significant for further and future revaluation. The gold standard of immunocytochemistry, in order to lead to an accurate diagnosis, is the use of a panel with at least two or three markers in combination with cytomorphology [17, 24]. Attention should be made to the extent, intensity, and pattern of staining. Liquid-based cytology has been validated extensively for the preoperative diagnosis of thyroid nodules [23, 24]. This method is fully reproducible, performed by minimum training of personnel, is safe, time effective, and offers the possibility of an accurate application of immmunocytochemistry for the study of neoplastic thyroid lesions. To our knowledge, immunocytochemistry in the diagnosis of papillary thyroid cancer in FNA's using liquid-based cytology is for the first time used in such an extensive material.

## 6. Conclusions

Our results suggest the following. (1) A precise diagnosis of PTC in fine needle aspiration material is practicable and credible with the use of contemporary liquid cytology techniques and immunocytochemistry, which is the key for a correct and accurate diagnosis. (2) Positive immunoexpression of CK-19, Galectin-3, CD-44, and HBME1 contributes the most in PTCs diagnosis, and these antibodies can be considered as first-line immunomarkers. Although HBME1 is not necessarily a marker of papillary differentiation, it serves as an indicator of thyroid papillary malignancy especially in combination with the above markers. (3) CD-56 can assist in decision making about the benign or malignant nature of the aspirated material. Loss of expression seems to agree with the presence of papillary thyroid cancer. (4) Loss of the expression of E-cadherin seems to serve as a good indicative marker of malignancy. (5) Thin layer cytology is a promising technique for the investigation of thyroid lesions and increasing the diagnostic accuracy in papillary carcinoma. (6) There is a clear need for the development of additional molecular markers in order to improve the diagnostic capabilities and thereby advance the clinical management in patients with borderline FNA results.

## Figures and Tables

**Table 1a:** (a) Results of Immunocytochemistry in FNAs, cases, and controls, (*n* is the number of samples)

	Cases		Cases			Controls	
Immunomarker	Expression		Loss of expression		*P*	Expression	
	*n*	%	*n*	%		*n*	%

CK-19	77	92.7	6	7.3	<.005	—	—
Galectin-3	74	89.1	9	10.9	<.005	—	—
HBME1	65	78.3	18	21.7	<.005	—	—
CD-44	72	86.7	11	13.3	<.005	—	—

**Table 1b:** (b) Expression of CD-56 and E-Cadherin in FNAs, cases, and controls. *n* is the number of samples

	Cases		Cases			Controls	
Immunomarker	Expression		Loss of expression		*P*	Expression	
	*n*	%	*n*	%		*n*	%

CD-56	3	3.6	80	96.4	<.005	30	100
E-Cadherin	5	6.0	78	94.0	<.005	30	100
